# Mechanism of immune infiltration in synovial tissue of osteoarthritis: a gene expression-based study

**DOI:** 10.1186/s13018-023-03541-x

**Published:** 2023-01-21

**Authors:** Qingyu Zhang, Chao Sun, Xuchang Liu, Chao Zhu, Chuncheng Ma, Rongjie Feng

**Affiliations:** Department of Orthopedics, Shandong Provincial Hospital Affiliated to Shandong First Medical University, No. 324, Road Jing Wu Wei Qi, Jinan, 250021 Shandong China

**Keywords:** Osteoarthritis, Synovial, Immune infiltration, Biomarkers, Weighed gene co-expression network analysis

## Abstract

**Background:**

Osteoarthritis is a chronic degenerative joint disease, and increasing evidences suggest that the pathogenic mechanism involves immune system and inflammation.

**Aims:**

The aim of current study was to uncover hub genes linked to immune infiltration in osteoarthritis synovial tissue using comprehensive bioinformatics analysis and experimental confirmation.

**Methods:**

Multiple microarray datasets (GSE55457, GSE55235, GSE12021 and GSE1919) for osteoarthritis in Gene Expression Omnibus database were downloaded for analysis. Differentially expressed genes (DEGs) were identified using Limma package in R software, and immune infiltration was evaluated by CIBERSORT algorithm. Then weighted gene co-expression network analysis (WGCNA) was performed to uncover immune infiltration-associated gene modules. Protein–protein interaction (PPI) network was constructed to select the hub genes, and the tissue distribution of these genes was analyzed using BioGPS database. Finally, the expression pattern of these genes was confirmed by RT-qPCR using clinical samples.

**Results:**

Totally 181 DEGs between osteoarthritis and normal control were screened. Macrophages, mast cells, memory CD4 T cells and B cells accounted for the majority of immune cell composition in synovial tissue. Osteoarthritis synovial showed high abundance of infiltrating resting mast cells, B cells memory and plasma cells. WGCNA screened 93 DEGs related to osteoarthritis immune infiltration. These genes were involved in TNF signaling pathway, IL-17 signaling pathway, response to steroid hormone, glucocorticoid and corticosteroid. Ten hub genes including MYC, JUN, DUSP1, NFKBIA, VEGFA, ATF3, IL-6, PTGS2, IL1B and SOCS3 were selected by using PPI network. Among them, four genes (MYC, JUN, DUSP1 and NFKBIA) specifically expressed in immune system were identified and clinical samples revealed consistent change of these four genes in synovial tissue retrieved from patients with osteoarthritis.

**Conclusion:**

A 4-gene-based diagnostic model was developed, which had well predictive performance in osteoarthritis. MYC, JUN, DUSP1 and NFKBIA might be biomarkers and potential therapeutic targets in osteoarthritis.

**Supplementary Information:**

The online version contains supplementary material available at 10.1186/s13018-023-03541-x.

## Introduction

Osteoarthritis is a chronic disease featured by the breakdown of articular cartilage and underlying bone, accompanied by progressive destruction of synovium, ligaments, supporting muscles and meniscus, acting as one of the leading causes of joint disability [1, 2]. Osteoarthritis affects an estimated 240 million people in the world [3], and the risk factors for this disease include age, gender, obesity, joint injury [4], abnormal mechanical load, inadequate nutritional supply and genetic factors [5, 6]. Although osteoarthritis can violate any joints, joints in the hands, knees, hips and spine are the most commonly affected [1–3]. The main symptom of osteoarthritis is joint pain, which is not evident at the early stage. Currently, drugs (e.g., paracetamol and naproxen) and physical therapeutic strategies (e.g., exercise and use of a cane) could only alleviate the pain and delay the progression of osteoarthritis to a certain extent [7]. For patients whose quality of life is significantly compromised, joint replacement is recommended. Searching for effective biomarkers is particularly important for the early diagnosis and treatment of osteoarthritis.

Increasing evidences support that osteoarthritis is not merely a cartilage wear-triggered mechanical disorder. The pathogenic mechanism involves the immune system and inflammation [5, 6, 8]. Imaging, pathological and clinical findings indicate that synovial changes occur earlier than the pathological degeneration of cartilage [9–11] and synovitis featured by the recruitment of immune cells exists in the whole developmental process of osteoarthritis [12, 13]. Infiltration of activated macrophages and subpopulations of T cells in synovial tissue has been closely linked to the initiation of osteoarthritis [14]. Moreover, the significantly increased T helper type 1 cell and inflammatory cytokines including interferon γ, and interleukin (IL)-2/-10 in synovial fluid contributed to the disease aggravation [15]. Therefore, it is unsurprising that immunological parameters of synovial tissue and dysregulation of gene expression act as crucial discriminants of osteoarthritis.

Immune infiltration as well as associated hub genes and regulatory mechanism has been a hot area of research in cancer [16–18]. However, hub genes related to immune infiltration in synovial tissue of osteoarthritis have not been fully identified. There were several studies [16–20] investigating this topic by bioinformatics analysis. For instance, Cai et al. revealed that 14 hub genes (e.g., CCL20, CD44, CX3CR1, CXCL2) related to chemokine and cytokine activity participated in the immune infiltration of osteoarthritis [16]. Hu et al. also conducted bioinformatics analysis and then demonstrated that after stimulation of IL-1β, the expression levels of TCA1, TLR7, MMP9, CXCL10, CXCL13, HLA-DRA and ADIPOQSPP1 were significantly higher in the IL-1β-induced group than in the control group by using in vitro experiments [19]. However, the molecular regulatory network for the hub genes was not constructed in these studies. Therefore, an updated investigation is imperative.

In this study, based on the integrated analysis of several datasets retrieved from the Gene Expression Omnibus (GEO) database, we selected the differentially expressed genes (DEGs) associated with synovial immune infiltration in osteoarthritis using weighted gene co-expression network analysis (WGCNA). Furthermore, several feature genes were uncovered by protein–protein interaction (PPI) analysis and genes tissue distribution and then confirmed by clinical samples.

## Methods

### Data acquisition and preprocessing

Totally four microarray datasets of osteoarthritis in the GEO database (https://www.ncbi.nlm.nih.gov/geo/) were acquired by using following keyword: osteoarthritis. Data in the GSE55457 dataset (synovial membrane samples from 10 osteoarthritis and 10 control subjects), GSE55235 dataset (synovial membrane samples from 10 osteoarthritis and 10 control subjects) and GSE12021 dataset (synovial membrane samples from 10 osteoarthritis and 10 control subjects) were generated on GPL96 platform. GSE1919 dataset (synovial membrane samples from 5 osteoarthritis and 10 control subjects) was generated on the GPL91 platform. Genes were annotated according to the annotation files provided by corresponding platforms. Probes matched no gene symbols were removed. For multiple probes matched to the same gene symbol, the mean value was used finally. The platform for GSE55457, GSE55235 and GSE12021 datasets was the same, and these three datasets were merged into one dataset for analysis after eliminating the batch effect using the ComBat function provided by the SVA package (version 3.340). GSE1919 dataset was applied as an external validation dataset.

### Differential expression analysis

Differentially expression analysis between osteoarthritis vs. normal control was conducted using a *t* test provided in the Limma package (version 3.10.3), followed by multiple test correction using Benjamini and Hochberg method. DEGs were selected with a cutoff of adjusted *p* value (adj. *p* val) < 0.05 and |log(Fold Change(FC))|> 0.585. The ggplot2 package and the pheatmap package were used to visualize the DEGs by drawing the volcano plot and heatmap.

### Evaluation of immune cells infiltration

Normalized gene expression data were used to infer the proportions of infiltrating immune cells. The relative abundance of 22 immune cells in each sample was evaluated by the CIBERSORT algorithm referring to the gene expression signature template provided on CIBERSORT official website (https://cibersortx.stanford.edu/, access on July 15, 2022). The parameter was set as perm = 100 and QN = F. The proportion of immune cells in each group was described in the heatmap. Pearson correlation analysis was performed to assess the associations among immune cells.

#### WGCNA

The calculation principle of WGCNA proposes that genes highly coexpressed in one module are a set of genes associated with the specific disease phenotype. We selected the genes with the absolute deviation of median expression ranked in the top 2000 of all the genes for WGCNA analysis. The adjacency matrix weight parameter power value was first determined to balance the relationship between mean connectivity and scale independence, and the intergenic diverging coefficients were then determined. Next, cluster dendrogram was built to assign genes into diverse modules with the dynamic mixed cutting method, and the minimum number of genes in the module was set as 30. The *p* value for each gene between groups was calculated using a *t* test, and the log *p* value for each gene was defined as gene significance (GS). Module significance (MS) was calculated by the mean value of GS. Finally, the module–trait relationships were computed to reveal the gene modules significantly associated with disease phenotype.

### Functional enrichment

Venn analysis was performed to manifest the common genes between DEGs and module genes, and these common genes were considered as key module genes. In order to explore their involved Kyoto Encyclopedia of Genes and Genomes (KEGG) pathways and gene ontology annotations, enrichment analysis was conducted utilizing clusterProfiler (version 3.16.0), with cutoff values of gene count ≥ 2 and *p* < 0.05. Results of functional enrichment analysis were visualized by the ggplot2 package.

### PPI network

The interactions among proteins encoded by key module genes were predicted on the basis of the Search Tool for the Retrieval of Interacting Genes (STRING) database (version 11.5, https://cn.string-db.org/, accessed on July 15, 2022), with PPI score set as 0.4 and species as human. The network was then constructed using Cytoscape software (version 3.6.1, Seattle, WA, USA) based on the predicted interactions. The topological property of nodes in the network was analyzed using the cytoHubba algorithm, including degree, maximal clique centrality (MCC), maximum neighborhood component (MNC), betweenness and closeness. The hub genes were defined by the intersection of the top 15 genes for each topological property.

### Tissue distribution of hub genes

To identify genes that specifically expressed in the immune system, the distribution of hub genes in different cells or tissues was explored on the basis of the BioGPS database (http://biogps.org/#goto=welcome, accessed on July 15, 2022). The genes specifically expressed in the immune system were considered as feature genes.

### Competing endogenous RNAs (ceRNA) network

The circRNA/lncRNA–miRNA–mRNA ceRNA network was established based on the ceRNA theory to screen circRNA, lncRNA and miRNA that can regulate the feature genes. The upstream miRNAs for feature genes were predicted using the miRWalk 3.0 tool (http://mirwalk.umm.uni-heidelberg.de/, access on July 15, 2022), and the miRNA–target pair existed in the miRDB database (http://www.mirdb.org/, accessed on July 15, 2022) with score = 1 was screened. The lncRNAs or circRNAs for miRNAs were predicted based on DIANA-LncBase v.2 (http://www.microrna.gr/LncBase/, accessed on July 15, 2022, score > 0.95, tissue: bone marrow) and ENCORI (https://starbase.sysu.edu.cn/, accessed on July 15, 2022) databases, respectively. The ceRNA network was established by integrating the predicted lncRNAs–miRNAs pairs, circRNAs–miRNAs pairs and miRNA–targets pairs by using Cytoscape.

### Validation of feature genes using GSE1919 dataset

The expression data of feature genes in the GSE1919 dataset were used as eigenvalues to construct the support vector machine (SVM) classifier using the e1071 SVM package with the parameters of sigmoid kernel and tenfold cross-validation. In addition, the expression of feature genes in the GSE1919 dataset was visualized using the ggplot2 and ggpubr packages.

### RT-qPCR

Synovial tissue samples were collected from three osteoarthritis patients receiving joint replacement and three normal controls receiving arthroscopic exploration in Shandong Provincial Hospital. All participants were informed consent, and this study was approved by the Ethics Committee of Shandong Provincial Hospital. The expression of feature genes was validated using RT-qPCR. Briefly, total RNA isolation from synovial tissues was conducted using TRIZOL reagent, and RNA concentration was determined on a microplate reader. The cDNA was synthesized firstly by reverse transcription with PrimeScript™RT Master Mix (TAKARA, Shiga, Japan). Next, real-time PCR was conducted with Power SYBR Green PCR Master Mix (Thermo, Waltham, MA, USA) on a PCR Amplifier.

## Results

### DEGs in synovial membrane tissue of osteoarthritis

**Fig. 1:**
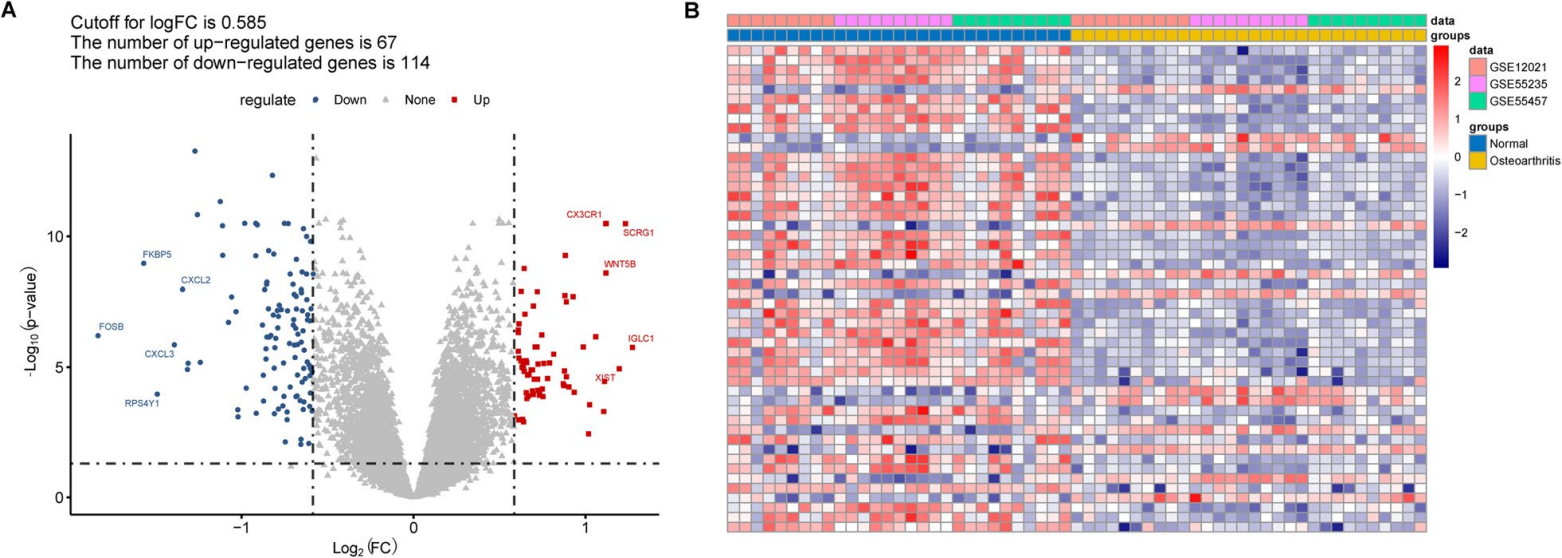
Differential expression analysis. **A** Volcano plot showing the differentially expressed genes between osteoarthritis vs. normal control; **B** Heatmap showing the expression pattern of genes in between osteoarthritis versus normal control

After eliminating the batch effect, GSE55457, GSE55235 and GSE12021 datasets were merged into one dataset. After normalization, the gene expression of samples in different datasets was at the same level (Figure S1A), and no significant separation among samples in three datasets (Figure S1B), indicating that the merged dataset could be used for subsequent analysis. Differential analysis discovered 181 DEGs between osteoarthritis vs. normal control, of which expression of 67 genes increased, while expression of 114 genes decreased in synovial membrane tissue of osteoarthritis patients than that of normal control (Fig. 1A). The expression pattern of these genes could distinguish samples from osteoarthritis to normal control (Fig. 1B).

### Immune cells infiltration in osteoarthritis synovial membrane tissue

**Fig. 2:**
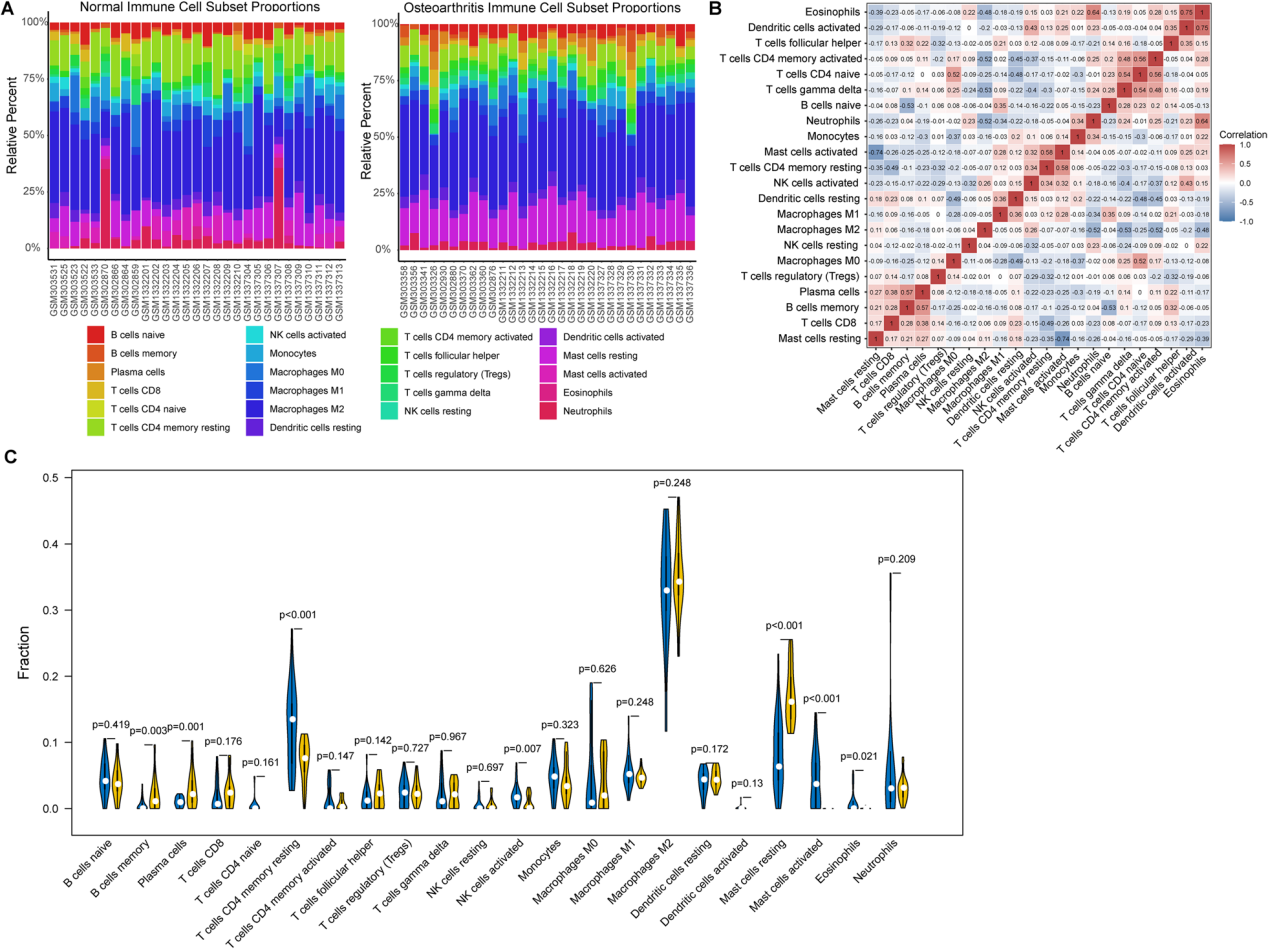
Evaluation of immune cells infiltration. **A** Landscape of immune cells infiltration in synovial membrane tissue of normal control (left) or osteoarthritis patients (right); **B** Correlation analysis among 22 immune cells in tumor samples; and **C** Violin plot showing the differences on infiltration abundance of 22 immune cells in synovial membrane tissue of normal control (blue) and osteoarthritis patients (yellow)

The relative infiltration abundance of 22 immune cells in synovial membrane tissue was evaluated for each sample. M2 macrophages, resting mast cells, memory CD4 T cells and B cells accounted for the majority of immune cell composition in synovial membrane tissue (Fig. 2A). Pearson correlation analysis revealed that eosinophils were significant correlated with activated dendritic cells (*r* = 0.75) and neutrophils (*r* = 0.64). Activated memory CD4 T cells showed strong positive correlations with naive CD4 T cells (*r* = 0.56) and gamma delta T cells (*r* = 0.48), while had strong negative correlations with M2 Macrophages (*r* =  − 0.52), resting dendritic cells (*r* =  − 0.45) and activated NK cells (*r* =  − 0.37) (Fig. 2B). Synovial membrane tissue in normal control had a high abundance of resting memory CD4 T cells (*p* < 0.001), activated NK cells (*p* = 0.007), activated mast cells (*p* < 0.001) and eosinophils (*p* = 0.021), while in osteoarthritis patients, the high infiltrating abundance of resting mast cells (*p* < 0.001), B cells memory (*p* = 0.003) and plasma cells (*p* = 0.001) were observed (Fig. 2C).

### Gene modules related to osteoarthritis and immune status

**Fig. 3:**
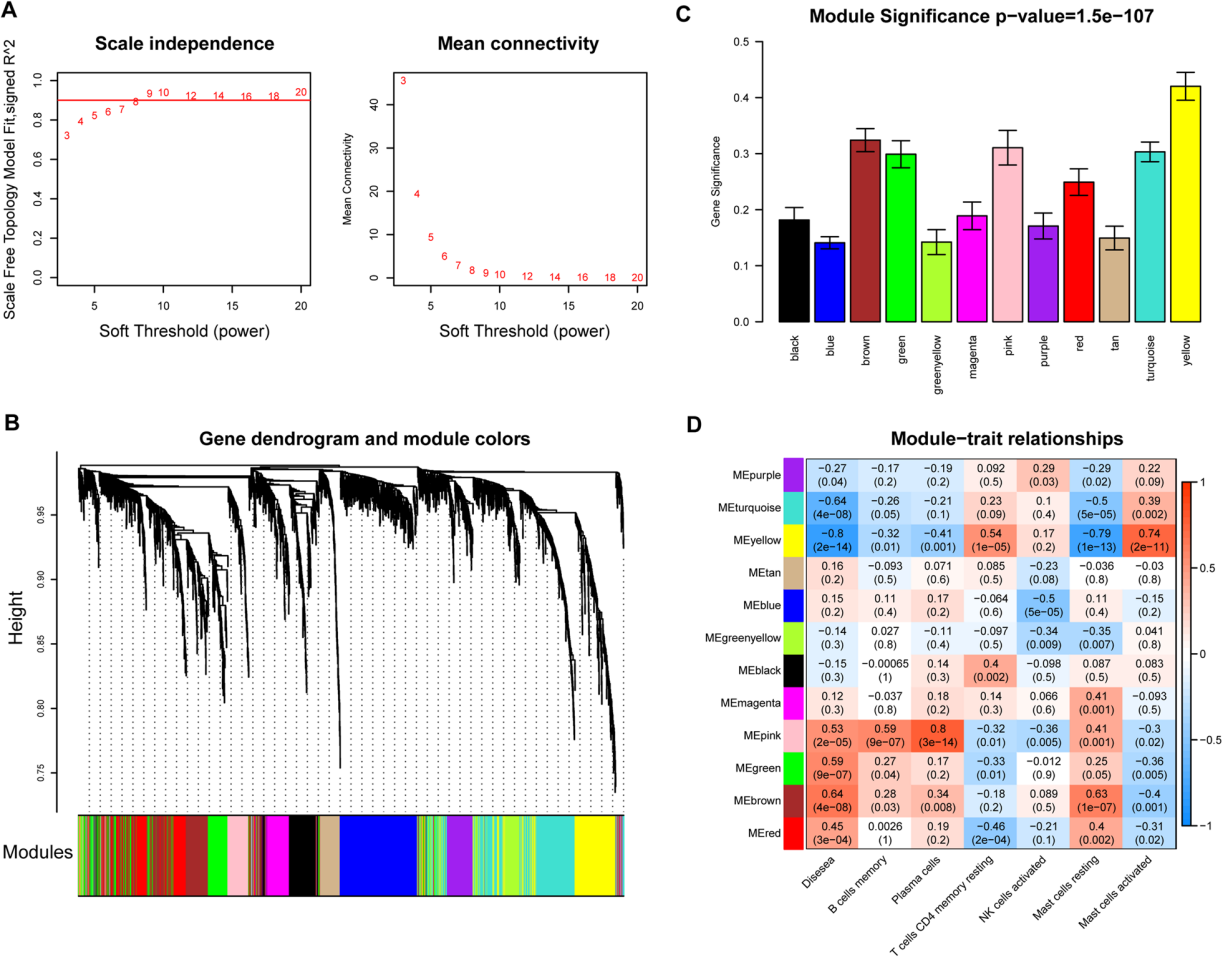
Weighed gene co-expression network analysis. **A** The scale independence (left) and mean connectivity (right) for various soft threshold powers; **B** Cluster dendrogram. Each color branch represents a color-coded module containing a highly interconnected set of genes; **C** Disease status correlated co-expression modules. *X*-axis represents different gene modules, and *Y*-axis represents the overall correlation coefficient between genes in each module and disease status; and **D** Module–trait relationships and *P* values for selected traits (osteoarthritis and immune cells)

The soft threshold power of 8 (no scale R2 = 0.9) in WGCNA was determined to construct an approximate scale-free network (Fig. 3A). Cluster dendrogram with dynamic mixed cutting method revealed 12 highly connected gene modules (Fig. 3B). Among which, yellow, pink, green and brown modules indicated strong correlations with osteoarthritis (Fig. 3C). Further module–trait relationships analysis showed that yellow and pink modules strongly correlated with both osteoarthritis and multiple immune infiltrating cells (Fig. 3D). Therefore, the genes in yellow and pink modules were considered as osteoarthritis and immune status associated genes.

### Identification of feature genes

**Fig. 4:**
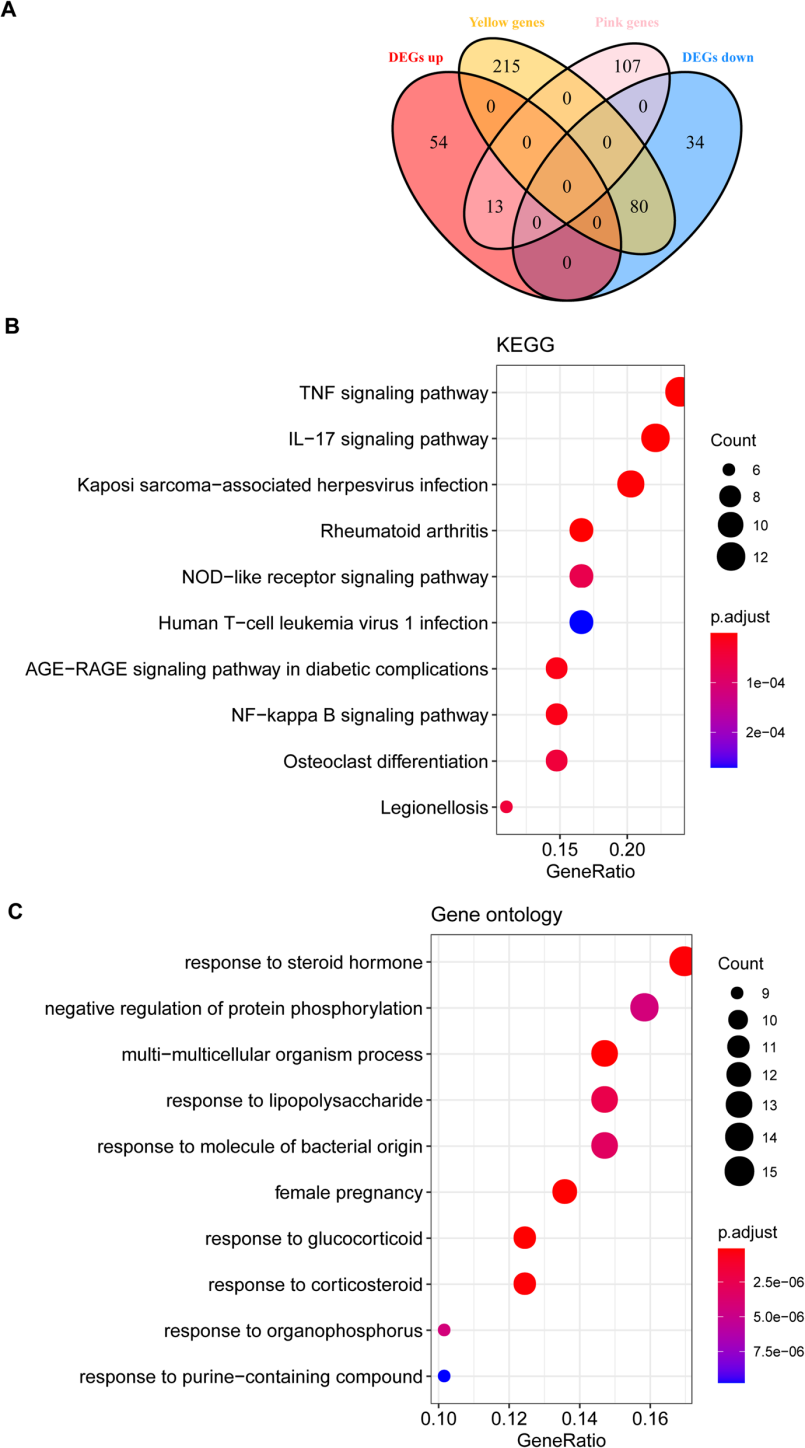
Functional enrichment analysis. **A** Venn diagram showing the common genes between gene modules and DEGs; **B** The significantly enriched KEGG pathways for common genes identified from Venn analysis; and **C** The significantly enriched biological processes terms for common genes identified from Venn analysis

**Fig. 5:**
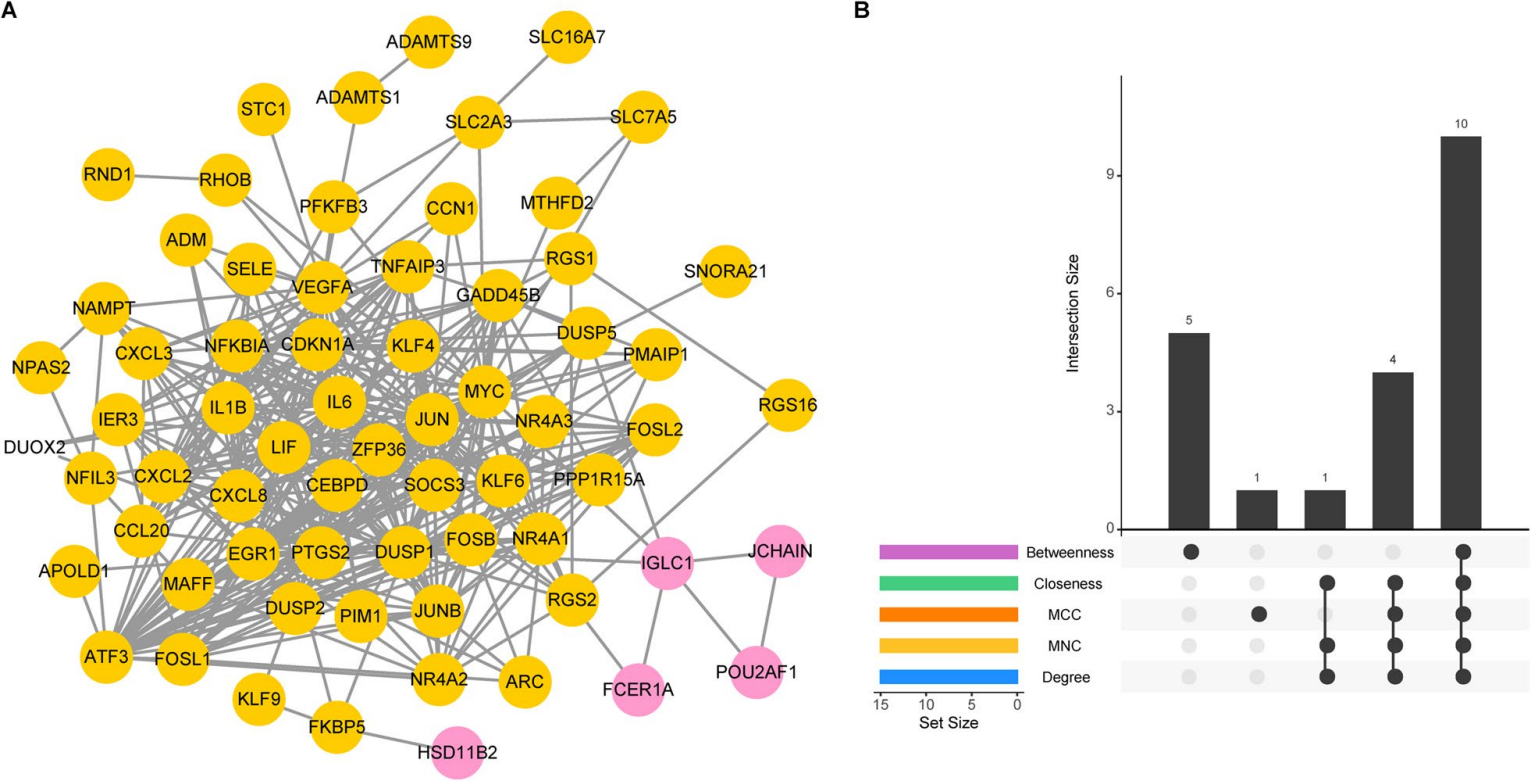
Protein–protein interaction network. **A** The protein–protein interaction network for common genes identified from Venn analysis. Pink nodes and yellow nodes represent the genes identified from pink or yellow modules in WGCNA; **B** Identification of hub genes from the network by cytoHubba algorithm

Venn analysis revealed a total of 93 common genes among two gene modules (yellow and pink) and DEGs (Fig. 4A), and these 93 genes were considered to involve in the occurrence and progression of osteoarthritis. Functional enrichment uncovered that these genes were implicated in TNF signaling pathway, IL-17 signaling pathway and rheumatoid arthritis pathways (Fig. 4B). Multiple biological processes were also enriched, including response to steroid hormone, response to lipopolysaccharide, response to glucocorticoid and response to corticosteroid (Fig. 4C). Based on the STRING database, the interactions among proteins encoded by these genes were analyzed, and 383 interactions involving 67 proteins were obtained (Fig. 5A). Ten hub genes in the intersection of five topological properties were determined (Fig. 5B, Table 1), including MYC, JUN, DUSP1, NFKBIA, VEGFA, ATF3, IL-6, PTGS2, IL1B and SOCS3. Among the ten hub genes, MYC, JUN, DUSP1 and NFKBIA were specifically expressed in immune system based on BioGPS database. Therefore, these four genes were considered as feature genes.

**Table 1:** The topological properties of top 10 genes in the PPI network

Genes	Betweenness	Closeness	Clustering coefficient	Degree	DMNC	EPC	MCC	MNC	Regulation
VEGFA	550.5598	43.83333	0.41667	24	0.60059	15.328	1.14E + 07	22	Down
IL1B	161.13187	44.83333	0.51077	26	0.65261	16.407	1.10E + 08	26	Down
IL-6	186.54771	46.33333	0.50246	29	0.66612	17.256	1.11E + 08	29	Down
ATF3	243.9095	47.41667	0.44153	32	0.60491	18.053	1.00E + 08	32	Down
PTGS2	183.68112	44.83333	0.50462	26	0.64475	16.824	1.03E + 08	26	Down
NFKBIA	195.74041	44.83333	0.53538	26	0.68406	16.762	1.10E + 08	26	Down
DUSP1	398.4935	46.83333	0.47537	29	0.6302	16.963	1.04E + 08	29	Down
SOCS3	138.20615	42.16667	0.65714	21	0.84748	15.408	1.10E + 08	20	Down
MYC	844.59928	49	0.35985	33	0.52481	17.376	6.37E + 07	32	Down
JUN	556.47519	50.5	0.37538	37	0.56523	18.421	1.12E + 08	36	Down

DMNC, density of maximum neighborhood component; EPC, edge percolated component; MCC, maximal clique centrality; and MNC, maximum neighborhood component

### Regulatory network for feature genes

**Fig. 6:**
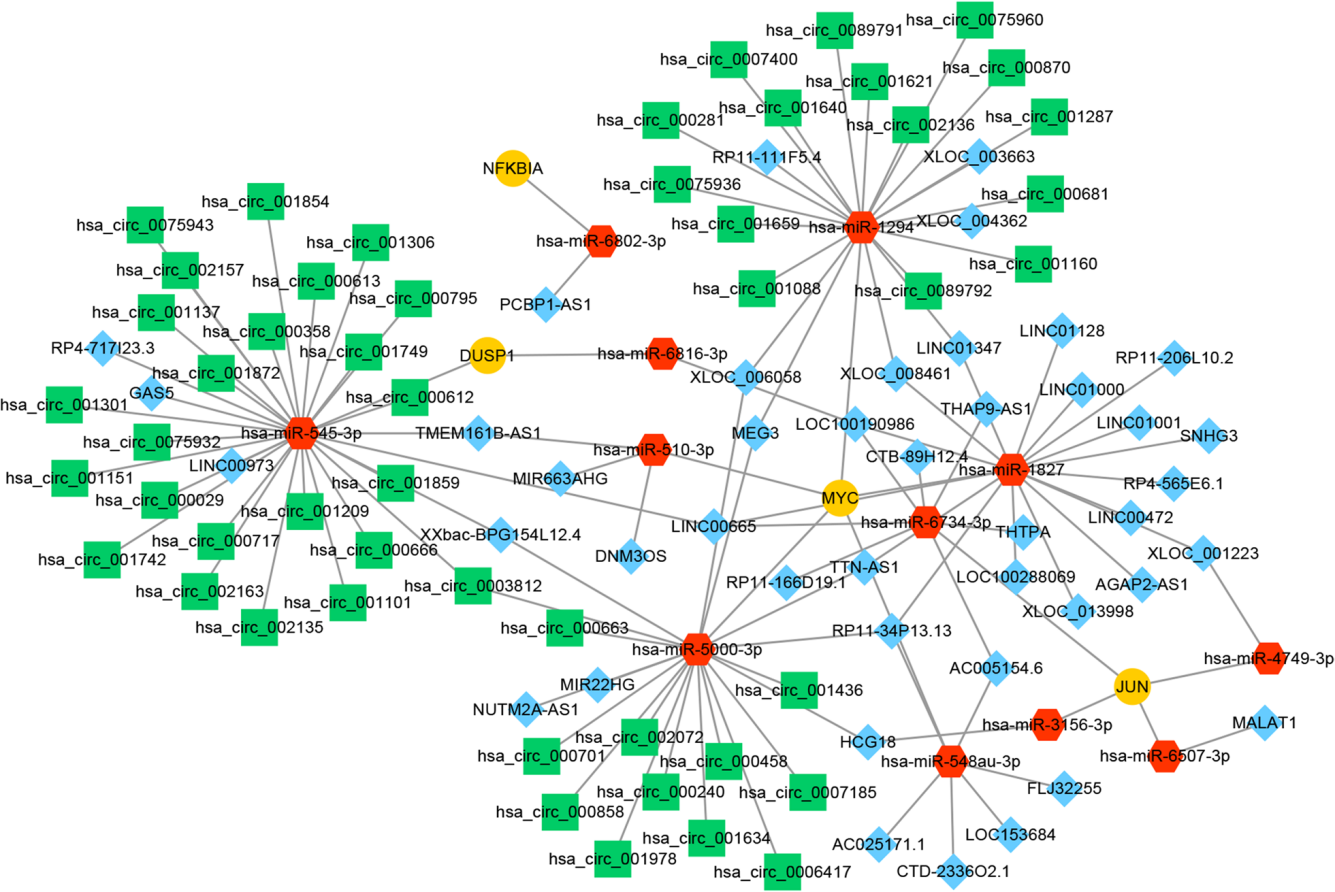
Regulatory network for feature genes. The lncRNA/circRNA–miRNA–target regulatory network. Yellow nodes represent feature genes; red nodes represent miRNAs; blue nodes represent lncRNAs; and green nodes represent circRNAs

The possible molecular regulation mechanism for feature genes was further explored. As shown in Fig. 6, the ceRNA network consisted of 122 interaction pairs involving 109 nodes, including 12 miRNAs, 43 lncRNAs and 50 circRNAs. For example, lncRNA PCBP1-AS1 might competitively bind to NFKBIA by sponging miR-6802-3p. The top 10 nodes are listed in Table 2, including seven miRNAs (e.g., miR-545-3p, miR-1294, miR-5000-3p), one lncRNA (LOC100190986) and two mRNAs (MYC and JUN). There were more interactions from circRNAs for miR-545-3p, miR-5000-3p and miR-1294, and more interactions from lncRNAs for miR-1827. MYC was targeted by five miRNAs (miR-510-3p, miR-5000-3p, miR-1827, miR-548au-3p and miR-1294). Four miRNAs (miR-6734-3p, miR-3156-3p, miR-6507-3p and miR-4749-3p) targeted JUN.

**Table 2:** The top 10 nodes in the ceRNA network

Type	Name	Degree	Betweenness	Closeness
miRNA	hsa-miR-545-3p	31	5068.856455	0.17252396
miRNA	hsa-miR-1294	23	3555.053968	0.16167665
miRNA	hsa-miR-5000-3p	21	4166.799978	0.17763158
miRNA	hsa-miR-1827	18	3665.771062	0.17940199
miRNA	hsa-miR-6734-3p	8	1413.341686	0.15606936
miRNA	hsa-miR-548au-3p	7	1013.228538	0.15789474
mRNA	MYC	5	2346.287224	0.17763158
mRNA	JUN	4	556.9764791	0.14361702
miRNA	hsa-miR-510-3p	4	649.2155012	0.16615385
lncRNA	LOC100190986	3	450.9923632	0.15835777

### Validation of feature genes using GSE1919 dataset

**Fig. 7:**
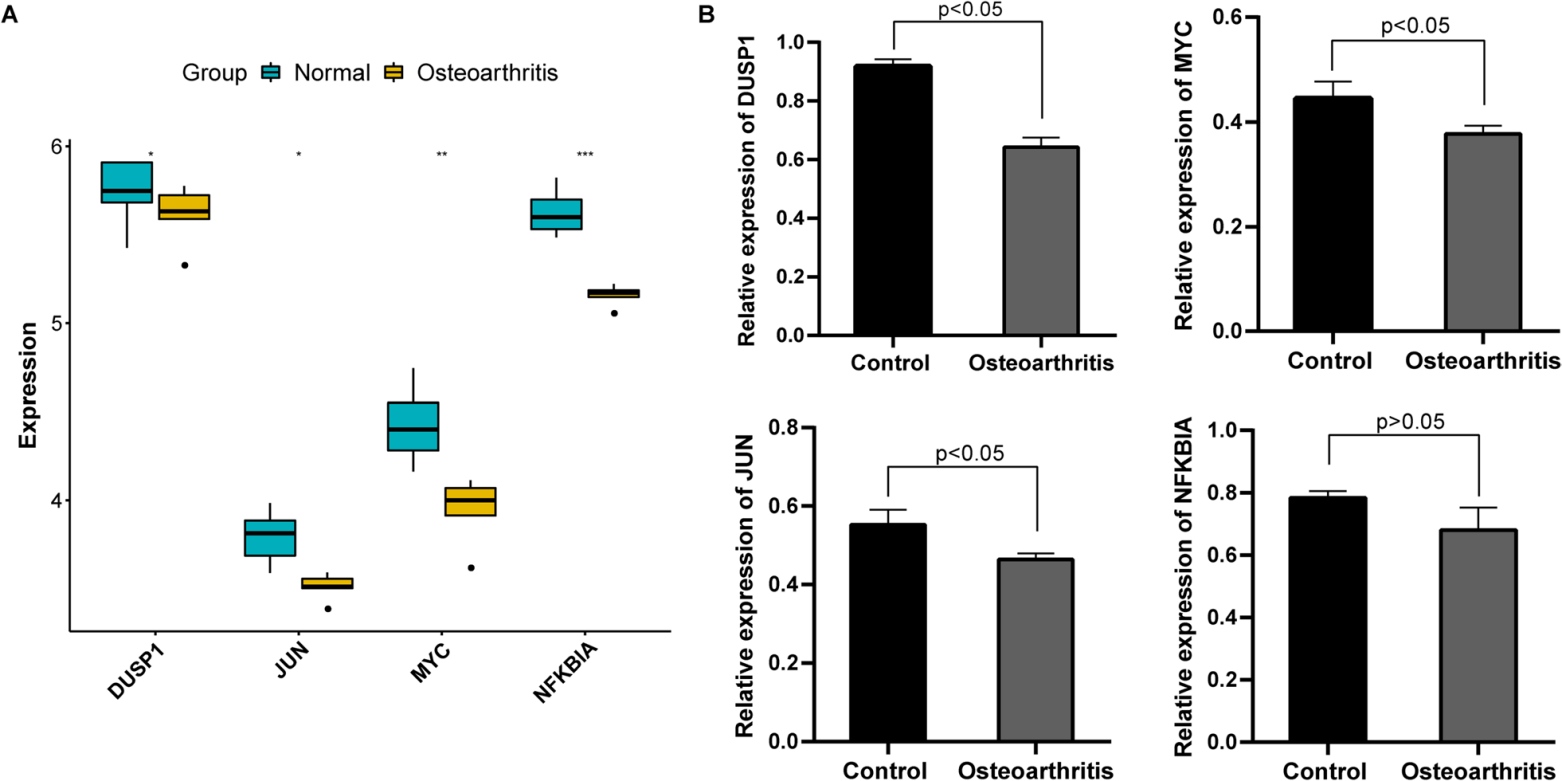
Validation of feature genes expression in GSE1919 dataset and clinical samples. **A** Boxplots showing the expression of feature genes in synovial membrane tissue of normal control and osteoarthritis patients. *P < 0.05; **P < 0.01; ***P < 0.01; **B** Expression of MYC, JUN and DUSP1 in synovial tissues of osteoarthritis patients and normal controls was determined by RT-qPCR. GAPDH was used as internal reference

The expression pattern of four feature genes in osteoarthritis and normal samples is presented in Fig. 7A. Compared to normal control, expression of MYC, JUN, DUSP1 and NFKBIA was down-regulated in osteoarthritis synovial membrane tissue (*p* value < 0.05).

### Expression of feature genes validated by RT-qPCR

For validating the differential expression of feature genes in synovial tissues of osteoarthritis and control subjects, RT-qPCR was performed (Fig. 7B). Consistently, we found that expression of MYC, JUN and DUSP1 was markedly reduced in synovial tissues of osteoarthritis patients than that of normal controls (*p* value < 0.05). Although there was no statistical significance, the expression of NFKBIA in synovial tissues of osteoarthritis patients showed a reduced trend in comparison with that of normal controls.

## Discussion

Diverse immune cells exert crucial roles in the initiation and progression of osteoarthritis. Prompt application of diagnostic and therapeutic biomarkers would dramatically improve the prognosis of osteoarthritic patients, but currently few molecular candidates fit the criteria of high sensitivity and specificity. In this study, we characterized the differences in terms of gene expression and immune cell infiltration in osteoarthritis synovial tissue compared to normal control. Further WGCNA screened 93 differentially expressed genes associated with osteoarthritis and immune status, and these genes were involved in immuno-inflammatory related pathways (e.g., TNF signaling pathway and IL-17 signaling pathway) and biological response processes (e.g., response to steroid hormone/glucocorticoid/corticosteroid). Finally, four feature genes, including MYC, JUN, DUSP1 and NFKBIA were identified, which had well predictive performance in osteoarthritis.

Immune infiltration evaluation indicated that macrophages, mast cells, memory CD4 T cells and B cells accounted for the majority of immune cell composition in synovial tissue. Osteoarthritis patients showed a relative high abundance of infiltrating resting mast cells, memory B cells and plasma cells. Increasing evidences have demonstrated that osteoarthritis is not non-inflammatory arthritis as previously described, but a disease that involves continuous low-level inflammation and activation of innate inflammatory pathways [21]. Synovial macrophages are the primary inflammatory and immune cells, and a large number of activated macrophages are found in synovial tissues of patients with osteoarthritis at different stages [22]. M1 macrophages account for the primary subgroup involving the inflammatory factors release, cartilage degradation and osteophyte formation in osteoarthritis, while M2 macrophages produce anti-inflammatory cytokines, showing a protective effect in osteoarthritis [22, 23]. The number of mast cells in synovium was markedly increased in osteoarthritic patients than control, and closely correlated with synovial inflammation and disease severity [24]. Elevated mast cell infiltration could amplify the inflammatory pathology and aggravate structural damage of joints in osteoarthritis [25, 26]. In addition, B cells and plasma cells were also detected in synovial tissue or synovial fluid of osteoarthritic patients, of which B cells were a source of IL-10, while plasma cells could secrete IL-6 [27, 28]. These findings suggested that targeting synovial immune cells might be a potential therapeutic strategy for osteoarthritic patients.

Based on WGCNA, we further revealed a set of genes associated with osteoarthritis and immune status, which were involved in the TNF signaling pathway, and IL-17 signaling pathway. Synovial cells can be divided into two types [29]. Type A includes macrophage-like cells with phagocytic function, while type B is fibroblast-like synoviocytes (FLS), which mainly provide lubrication and nutrition for joints by secreting hyaluronic acid [29, 30]. TNF-α could induce the production of various cytokines (matrix metalloproteinases (MMPs) and IL-34) to participate in the inflammatory response of osteoarthritis by activating different signals [31–34]. IL-17 is a pleiotropic inflammatory cytokine involved in osteoarthritis, and blocking the IL-17 signaling pathway can effectively relieve pain for patients [35, 36]. IL-17-mediated inflammation leads to mitochondrial dysfunction of FLS, which in turn stimulates the infiltration of inflammatory lymphocytes and autophagic apoptosis of FLS [37]. In the osteoarthritis mice model, IL-17 facilitates cartilage destruction and nociceptive properties by regulating inflammatory mediators [38]. In addition, these DEGs were also enriched in response to steroid hormone, glucocorticoid and corticosteroid. Glucocorticoids have been used for the treatment of osteoarthritis [39, 40]. These findings suggested that the screened genes played important roles in osteoarthritis.

Next, by further analysis of ten hub genes (MYC, JUN, DUSP1, NFKBIA, VEGFA, ATF3, IL-6, PTGS2, IL1B and SOCS3), we discovered that MYC, JUN, DUSP1 and NFKBIA were specifically expressed in the immune system and selected as biomarkers and potential therapeutic targets in osteoarthritis. Inhibition of the MYC gene promoted cell proliferation and repressed cell apoptosis, and expression of MMP-13, IL-6 and TNF-α in IL-1β-induced rat chondrocytes [41]. JUN has been reported to stimulate the apoptosis of chondrocytes by increasing the level of PUMA (a pro-apoptotic factor) [42] and BIM [43], serving an important role in osteoarthritis. Jian et al. demonstrated that expression of DUSP1 was reduced in synovial tissues facet joint osteoarthritis patients and IL-1β-induced FLS, and overexpression of DUSP1 inhibited the proliferation and inflammatory response in FLS [44]. DUSP1 has a protective effect on osteoarthritis by intercepting the expression of MMP-13 and activating the MAPK pathway [45]. NFKBIA is a member of the NFKB inhibitor family, which prevents inflammatory responses-related NFKB/REL complexes by interacting with REL [46]. NFKBIA was involved in the inflammatory effect of FLS, contributing to an elevated risk of hip osteoarthritis [46, 47].

LncRNA, circRNA and miRNA are non-coding RNAs (ncRNAs) that constitute approximately 90% of the RNA transcripts of the human genome. Despite lacking protein-coding potential, ncRNAs participated in a variety of pathological processes of osteoarthritis by regulating gene transcription and protein translation, serving as biological targets in the prevention, diagnosis and treatment of osteoarthritis [48, 49]. Therefore, the lncRNA/circRNA–miRNA–mRNA regulatory network for these four hub genes was constructed and several hub ncRNAs such as miR-545-3p, miR-1294 and miR-510-3p were selected. Liu et al. have demonstrated the effect of miR-510-3p in inhibiting the progression of osteoarthritis [50]. MiR-545-3p and miR-1294 have not been investigated in osteoarthritis, but studies have confirmed their effectiveness in regulating inflammation. For example, the dysregulation of miR-545 is associated with elevated expression of inflammatory cytokines and abnormal activation of macrophage in cirrhosis [51]. Besides, miR-545 could affect the inflammatory response in cardiomyocytes by targeting CXCL16 [52]. Upregulation of miR-1294 inhibits inflammation in atopic dermatitis by suppressing NF-κB pathway activation [53] and promotes the development of autoimmune disorders by regulating the release of pro-inflammatory cytokines [54].

Despite these findings, there remain some limitations in this study. First, only qPCR was performed to validate the gene expression at the transcriptomic level for three samples in each group. In addition, the biological function and the clinical correlations of these feature genes in the development of osteoarthritis have not been investigated. Second, the diagnostic value of these feature genes was only verified based on a small dataset, which undermines the power of the validation. Last but not least, the hub lncRNAs/circRNAs/miRNAs were predicted by using multiple bioinformatics analysis methods, but the role of these identified ncRNAs in the progression of osteoarthritis remains unclear. It should be admitted that the experimental validation section of this investigation is very elementary. However, findings of the bioinformatics analysis provide multiple directions for research about the pathological mechanism of osteoarthritis, which will be tapped in the future.

## Conclusion

In conclusion, gene expression and immune infiltration in osteoarthritis synovial tissue were characterized in this study. A set of genes related to osteoarthritis immune infiltration was identified, which might play essential roles in the development and progression of osteoarthritis. Four genes, MYC, JUN, DUSP1 and NFKBIA, were selected as potential diagnostic biomarkers in osteoarthritis. This study provided novel clues for the early diagnosis and treatment of osteoarthritis.

## Supplementary Information

**Additional file 1: Figure S1**. Principal component analysis and normalization of samples. **A** The distribution of expression of 27 samples involving principal component analysis (PCA) for confirming biological variability between different samples; **B** The distribution of expression of 30 samples before and after normalization.

## Data Availability

All data analyzed during this study are included in this published article.

## Abbreviations

GEO: Gene Expression Omnibus
WGCNA: Weighted gene co-expression network analysis
PPI: Protein–protein interaction
DEGs: Differentially expressed genes
FC: Fold change
GS: Gene significance
MS: Module significance
KEGG: Kyoto Encyclopedia of Genes and Genomes
MCC: Maximal clique centrality
MNC: Maximum neighborhood component
SVM: Support vector machine
