# Supplementary figures and images for: Mechanism of immune infiltration in synovial tissue of osteoarthritis: a gene expression-based study
Source: J Orthop Surg Res. 2023 Jan 21;18:58. doi: 10.1186/s13018-023-03541-x (PMC9862811; doi:10.1186/s13018-023-03541-x)

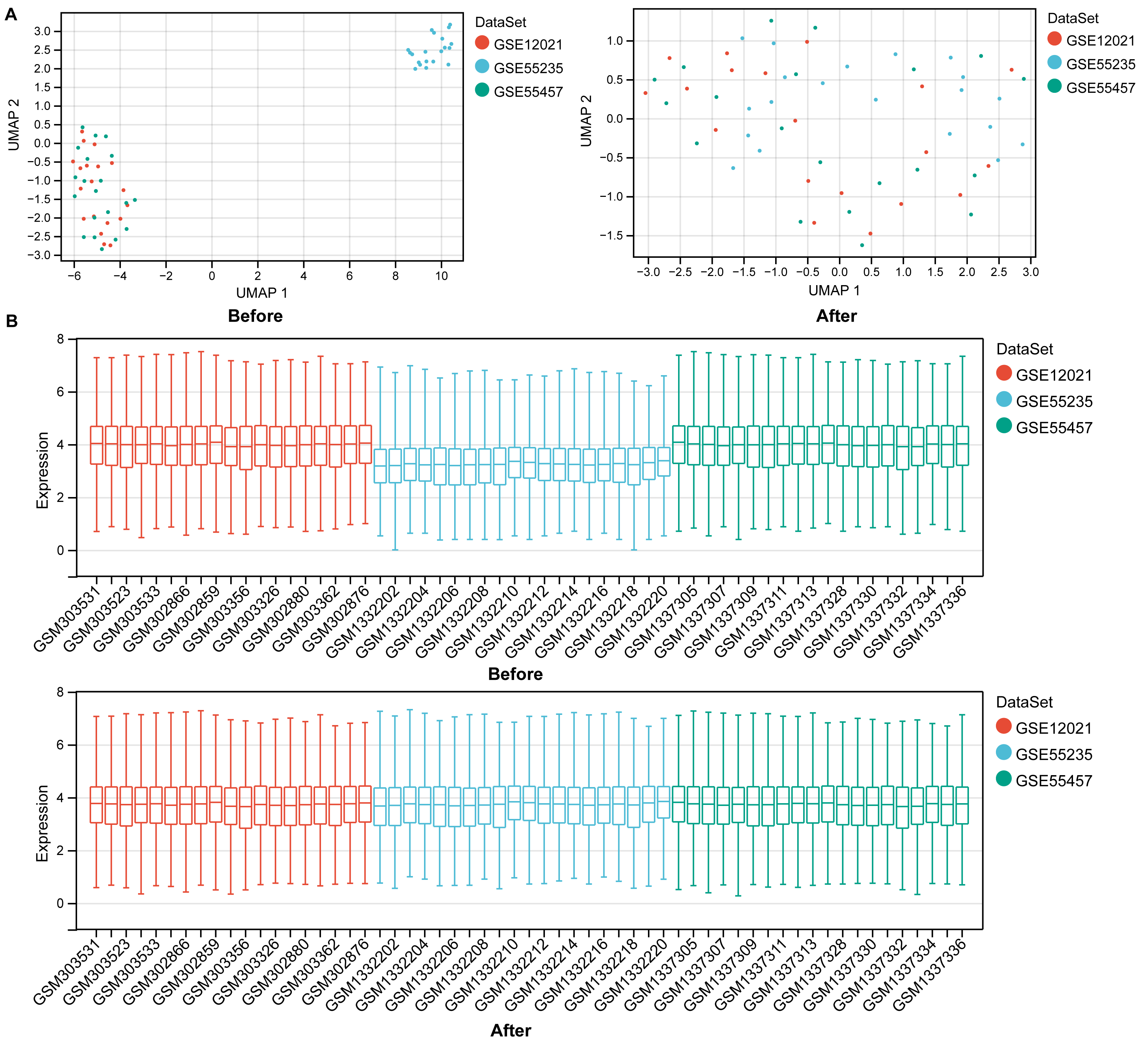

Supplement: Supplementary file 1 — Additional file 1: Figure S1. Principal component analysis and normalization of samples. A The distribution of expression of 27 samples involving principal component analysis (PCA) for confirming biological variability between different samples; B The distribution of expression of 30 samples before and after normalization. [file 13018_2023_3541_MOESM1_ESM.tif]
